# Long-term effects of warm water immersion on kidney tissue damage in diabetic rats

**DOI:** 10.22038/IJBMS.2024.74307.16141

**Published:** 2024

**Authors:** Faezeh Jozi, Nejat Kheiripour, Maryam Akhavan Taheri, Gholamreza Ghavipanjeh, Zahra Nasehi, Mohammad Esmaeil Shahaboddin

**Affiliations:** 1 Institute for Basic Sciences, Research Center for Biochemistry and Nutrition in Metabolic Diseases, Kashan University of Medical Sciences,; 2 Kashan, Iran; 3 Gametogenesis Research Center, Institute for Basic Sciences, Kashan University of Medical Science, Kashan, Iran; 4 Institute for Basic Sciences, Physiology Research Center, Kashan University of Medical Sciences, Kashan, Iran; 5 Department of Clinical Biochemistry, Faculty of Medicine, Kashan University of Medical Sciences, Kashan, Iran

**Keywords:** Diabetic nephropathies, Heat-shock proteins (HSP70), Hydrotherapy, Insulin, Oxidative stress

## Abstract

**Objective(s):**

This study aimed to investigate the effects of Warm Water Immersion (WWI) on inflammation, kidney function, and kidney tissue damage in rats with diabetes mellitus (DM).

**Materials and Methods:**

Forty male rats were divided into four groups: Healthy Control (HC), Diabetic Control (DC), Diabetic Rats treated with WWI (DW), and Healthy Rats treated with WWI (HW). Daily 15-minute WWI sessions at 43 °C were administered for eight weeks. Various parameters including lipids, fasting blood sugar (FBS), HbA1C, insulin, advanced glycation end products (AGEs), HSP70, glomerular filtration rate (GFR), urinary albumin excretion, creatinine, blood urea nitrogen (BUN), oxidative stress, anti-oxidant parameters, and gene expression of RAGE, VEGF, and TGFß1 were assessed. Histological examination of kidney tissue was also conducted.

**Results:**

Significant reductions in FBS, AGEs, glutathione, superoxide dismutase (SOD), and nitric oxide (NO) levels were observed in the DW group compared to DC. Expression of RAGE, VEGF, and TGFß1 genes decreased in DW. Triglycerides, total cholesterol, and LDL cholesterol were lower in DW. Insulin, HDL cholesterol, catalase, total anti-oxidant capacity (TAC), and tissue HSP70 were higher in DW. Histological assessment revealed reduced kidney damage in DW compared to DC.

**Conclusion:**

WWI for eight weeks shows promise in mitigating diabetic nephropathy in rats, suggesting its potential as a non-invasive adjunctive therapy for managing diabetes complications.

## Introduction

Diabetes mellitus (DM) stands as a widespread chronic metabolic condition marked by elevated blood sugar levels, increased lipid levels, oxidative stress, and heightened levels of inflammatory cytokines (1). Glucose interaction with biomolecules leads to advanced glycation end product (AGE) formation, altering the structure and function of nucleic acids, proteins, and lipids (2, 3). This process is associated with increased levels of receptors for AGEs (RAGE), activating a cascade that includes the generation of harmful oxygen radicals and consequent oxidative damage (4-6).

RAGEs act as intermediaries for inflammatory gene expression, impacting diverse cellular communication pathways, including the activation of the NF-κB transcription factor. This cascade contributes to cellular responses like inflammation, fibrosis, coagulation, vasculogenesis, and altered expression of adhesive molecules, cytokines, and growth factors (7-12).

The complications of DM are diverse, affecting cardiovascular health, causing nephropathy, neuropathy, retinopathy, and impairing wound healing. Diabetic nephropathy (DN) is a serious consequence, characterized by excessive glomerular filtration, proteinuria, and renal failure. Notably, the presence of urine casts, including hyaline and albumin casts, serves as a crucial indicator for assessing kidney function in DN (13, 14).

Effective strategies to manage DN include limiting the formation of AGE, impeding the interaction between AGEs and their receptor (RAGE), reducing the expression of RAGE, and enhancing the expression of heat shock proteins (HSPs), known as molecular chaperones (15, 16). HSPs play a vital role in stabilizing protein structure, reducing aggregation, preventing inappropriate interactions among cellular proteins, inhibiting protein glycation, and ameliorating DM-associated complications (17-20).

In diabetes, increased AGE levels lead to elevated RAGE expression. Extracellular AGEs interacting with RAGE trigger intracellular events, including the up-regulation of inflammatory genes. Conversely, extracellular HSP70 can engage with RAGE, preventing the interaction of AGEs with RAGE and mitigating complications associated with diabetes (21, 22). Previous studies highlight the critical role of HSPs, reporting a decrease in their expression in rats with type 1 DM and insulin-resistant diabetic patients (23, 24).

Warm water immersion (WWI) emerges as a potentially effective technique for inducing HSP expression and reducing oxidative stress and inflammation (25). While a study reported increased expression of the HSP72 gene and reduced proinflammatory cytokine levels in rats with acute pancreatitis subjected to WWI (26), conflicting findings exist, indicating a reduction in the expression of the HSP70 gene in healthy rats following regular fifteen-minute daily WWI for one week (27).

Despite these insights, the effects of WWI on DN and its mechanisms remain poorly understood. The objective of this study is to evaluate how WWI affects inflammation, kidney function, and tissue damage in streptozotocin (STZ)-induced diabetic rats.

## Materials and Methods

***Moral deliberations***

The research has obtained ethical clearance from the Ethics Review Board at the Kashan University of Medical Sciences. All methods of the study were in accordance with the ethical guidelines of this university.

**Figure 1:**
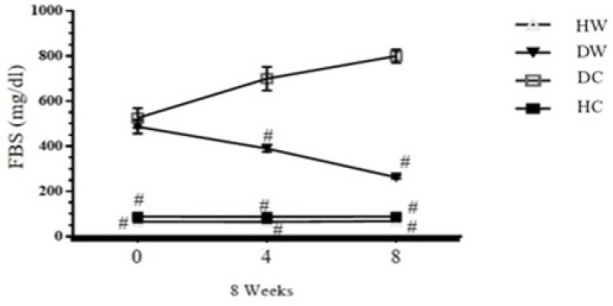
Effects of WWI on the level of FBS (Mean±SD) among diabetic WWI-treated (DW) rats compared to control groups

The levels of FBS were decreased in the diabetic WWI-treated (DW) group compared to the diabetes control (DC) group

HC: healthy control group; DC: diabetes control group; DW: diabetic WWI-treated group; HW: healthy WWI-treated group; FBS: fasting blood sugar.

#: Significant difference observed at *P*<0.001

**Figure 2:**
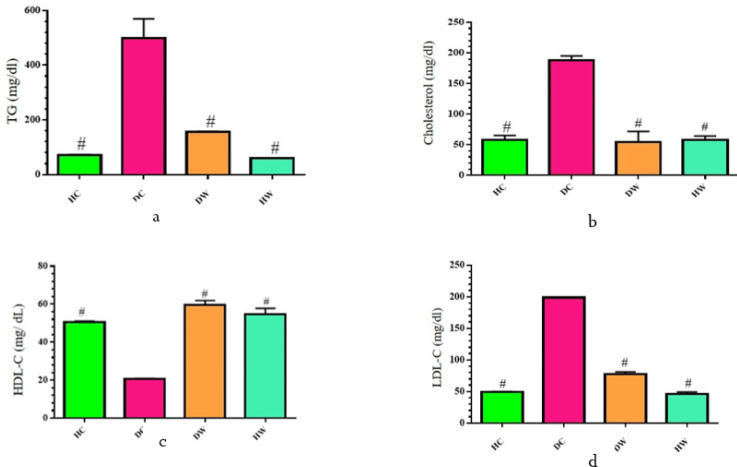
Effects of WWI on the serum lipid profile among diabetic WWI-treated (DW) rats compared to control groups

(a) TG, (b) Cholesterol, (c) HDL-c, and (d) LDL-c (Mean±SD)

HC: healthy control group; DC: diabetes control group; DW: diabetic WWI-treated group; HW: healthy WWI-treated group; TG: triglycerides; HDL-c: high-density lipoprotein- Cholesterol; LDL-c: low-density lipoprotein- Cholesterol. #: Significant difference with the DC group at a level of less than 0.001.

**Figure 3:**
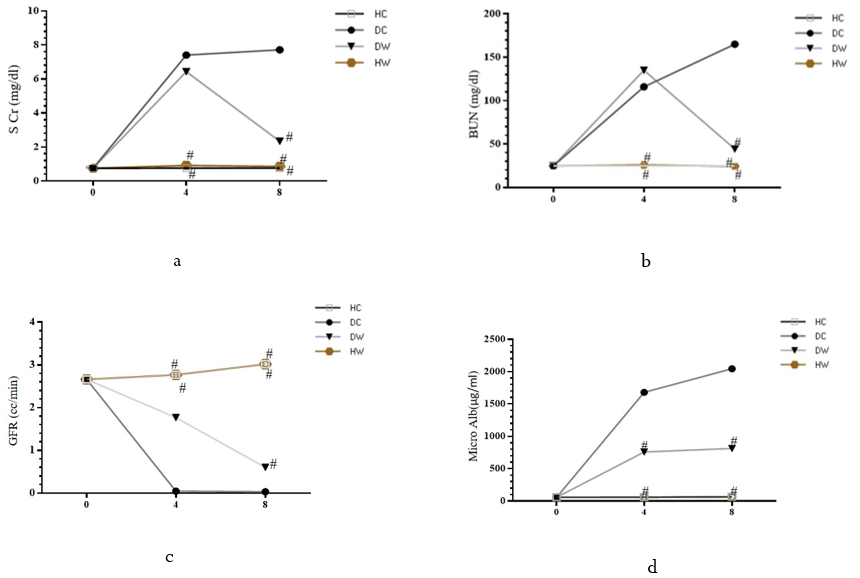
Effects of WWI on kidney function parameters after 4 and 8 weeks of treatment among diabetic WWI-treated (DW) rats compared to control groups

(a) SCr, (b) BUN, (c) GFR, and (d) Microalbumin (Mean±SD). HC: healthy control group; DC: diabetes control group; DW: diabetic WWI-treated group

HW: healthy WWI-treated group; SCr: serum Creatinine; BUN: blood Urea Nitrogen; GFR: glomerular filtration rate. #: Significant difference with the DC group at a level of less than 0.001

**Figure 4:**
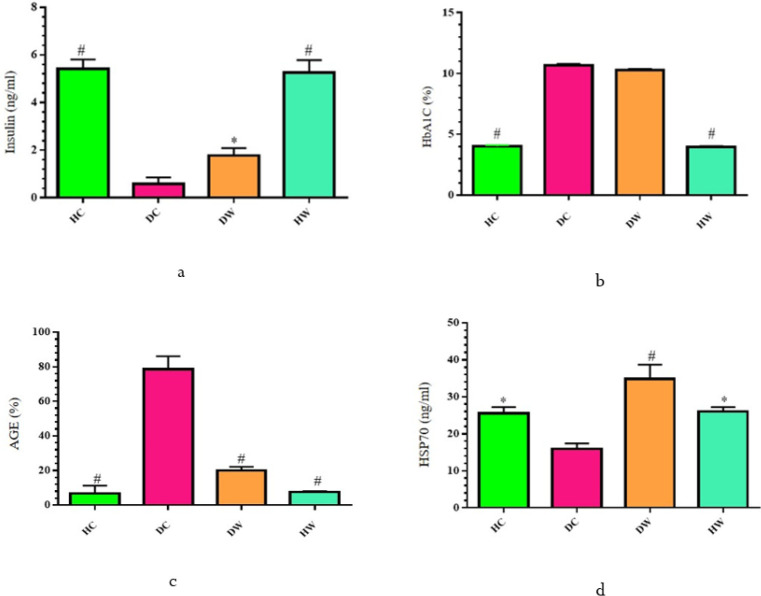
Effects of WWI on the mean scores for serum insulin, AGEs, HbA1C, and HSP70 levels among Diabetic WWI-treated (DW) rats compared to control groups

(a) Insulin, (b) HbA1C, (c) AGEs, and (d) HSP70 (Mean±SD). HC: healthy control group; DC: diabetes control group; DW: diabetic WWI-treated group; HW: healthy WWI-treated group; AGEs: advanced glycation end products; HbA1C: hemoglobin A1C; HSP70: heat shock protein 70. *: Significant difference with the DC group at a level of less than 0.01. #: Significant difference with the DC group at a level of less than 0.001.

**Figure 5:**
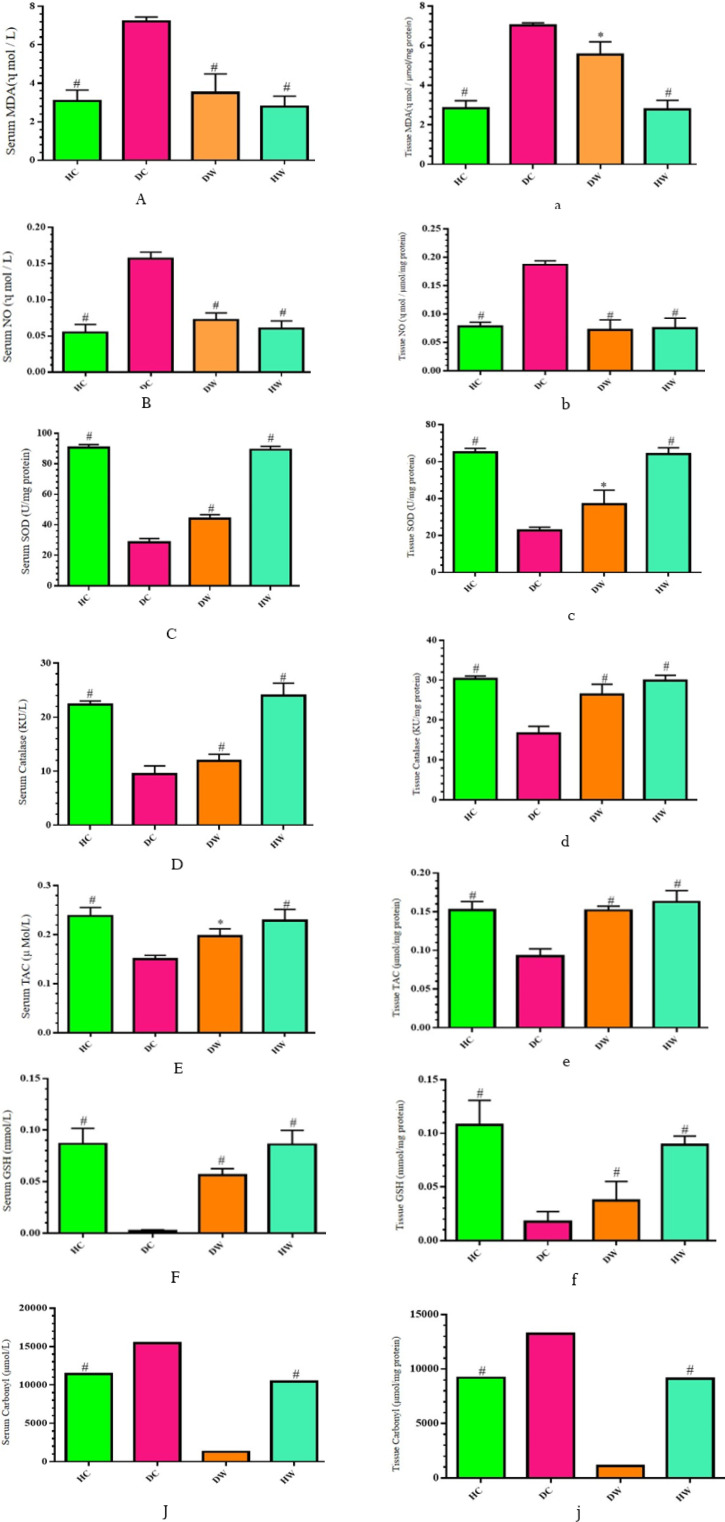
Effects of WWI on the mean scores for serum and tissue oxidative stress factors among diabetic WWI-treated (DW) rats compared to control groups

(A, a) MDA, (B,b) NO, (C,c) SOD, (D,d) Catalase, (E,e) TAC, (F,f) GSH, and (J,j) Protein Carbonyl (Mean±SD). HC: healthy control group

DC: diabetes control group; DW: diabetic WWI-treated group; HW: healthy WWI-treated group; MDA: malondialdehyde, NO: nitric oxide, SOD: superoxide dismutase, TAC: total anti-oxidant capacity, GSH: glutathione

*: Significant difference with the DC group at a level of less than 0.01, #: Significant difference with the DC group at a level of less than 0.001

**Figure 6:**
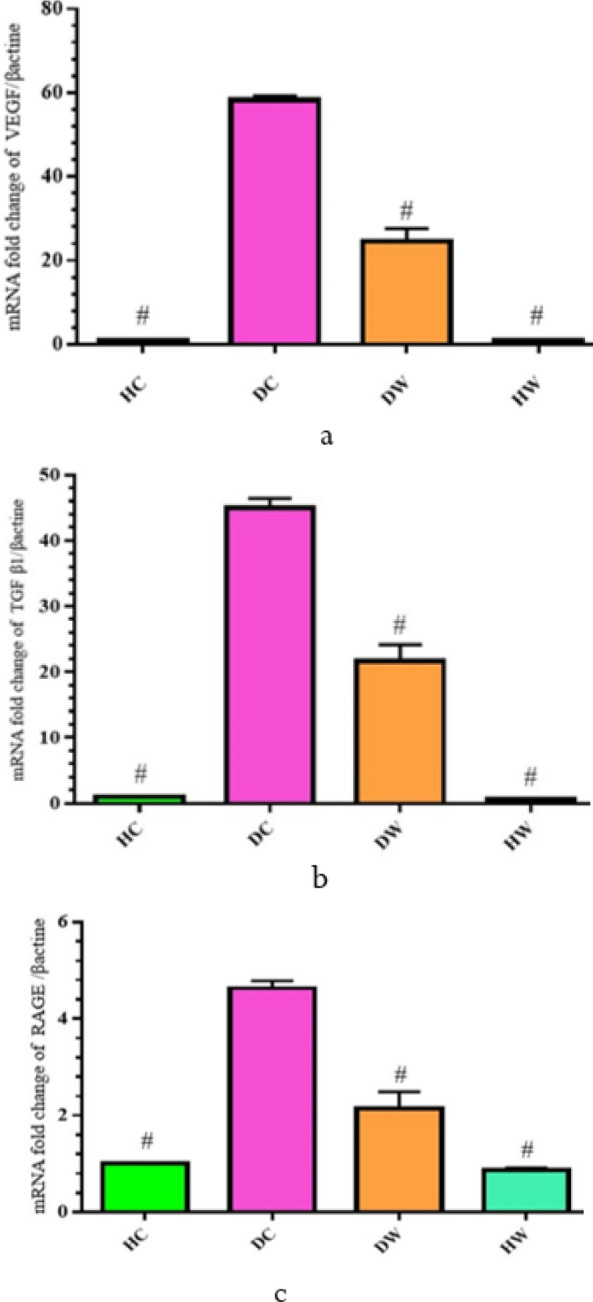
Eﬀects of WWI on the genes expression of (a) VEGF, (b) TGFß1, and (c) RAGE (Mean±SD)

Expression of these genes decreased in the diabetic WWI-treated group (DW) compared to the diabetic control group (DC)

HC: healthy control group; DC: diabetes control group; DW: diabetic WWI-treated group; HW: healthy WWI-treated group; RAGE: receptor of advanced glycation end product; VEGF: vascular endothelial growth factor; TGFß1: transforming growth factor ß1.

#: Significant difference observed at *P*<0.001.

**Figure 7:**
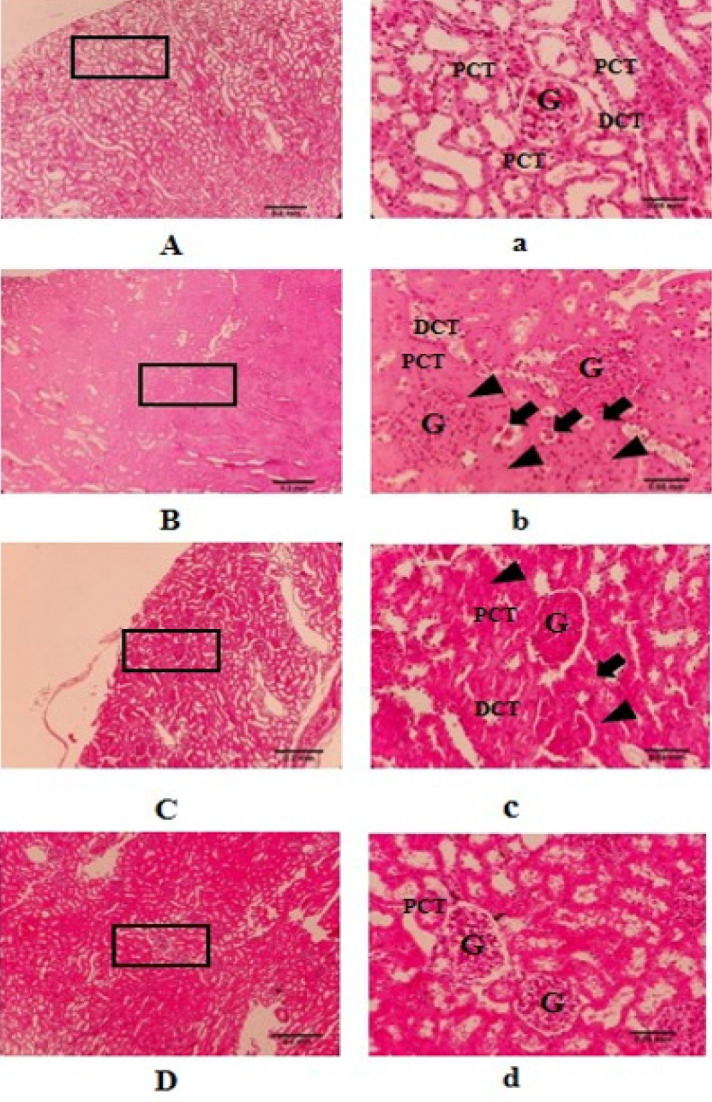
Eﬀects of WWI on the histological morphology changes of the kidney in diabetic rats

Panels A to D depict different experimental groups at magnifications X100 and X400. Healthy control (HC) group (A, a) displays healthy kidney morphology, while the diabetic control (DC) group (B, b) shows urinary tube swelling and hyaline cast presence. Diabetic WWI-treated (DW) group (C, c) exhibits similar changes but with less severity compared to the DC group. WWI-treated healthy (HW) group (D, d) mirrors the HC group's healthy kidney structure. The mean tissue damage score in the DW group was lower than the DC group, although not statistically significant (*P*>0.05).

**Table 1:** Group comparisons respecting the scores of kidney histological analysis among experimental groups

Group	HC Mean±SD	DC Mean±SD	DW Mean±SD	HW Mean±SD
Score				
Renal cortex	0±0 #	3.25±0.47	2.6±0.002	0±0 #

HC: healthy control group; DC: diabetes control group; DW: diabetic WWI-treated group; HW: healthy WWI-treated group.

#: Significant difference with the DC group at a level of less than 0.001

***Animals and intervention***

Forty male Wistar rats, averaging 230±30 grams in weight, were kept in controlled light/dark cycles and temperature for three weeks. Subsequently, they were randomly divided into four groups, each consisting of ten rats: a healthy control (HC) group, a diabetic control (DC) group, a diabetic WWI-treated (DW) group, and a healthy WWI-treated (HW) group.

Rats in the DC and the DW groups were initially given an intraperitoneal injection of STZ (65 mg per kilogram) (25). The injection was administered again if their fasting blood sugar (FBS) was below 15 mMol/L after 3–4 days, and rats with an FBS exceeding 15 mMol/L were enrolled in the study (28). Rats in the HC and the HW cohorts were administered a vehicle injection.

One week following the induction of DM and identification of hyperglycemia (denoted as time point zero in figures and tables), a daily fourteen-minute warm water immersion (WWI) regimen commenced for rats in the DW and HW categories, conducted at a temperature of 43 °C. During WWI, rats in these groups were put in a cage and the cage was put in a bath with circulating water. The intervention was daily implemented for eight weeks. During the WWI intervention, rats were restrained in order to prevent the potential confounding effects of physical exercise. Rats in the HC and the DC groups were kept at room temperature (25 °C) for the same period. Capillary tubes were utilized to collect eye blood samples at the conclusion of the fourth week of the intervention. Furthermore, urine samples were obtained at the beginning, middle, and end of the eight-week study period by isolating each rat in an individualized metabolic monitoring set up for six hours. Upon completion of the eight-week intervention, all rats underwent anesthesia using ether, and a heart blood sample was collected using a 25 Gx 1 needle. Subsequently, the rats were euthanized via aortic dissection. Then, kidney tissue was obtained through surgery and kept at –80 °C for further analysis. Blood samples were kept for the measurement of hemoglobin A1C (HbA_1C_). For further analysis, serum samples were obtained by centrifuging blood samples at 500 g for fifteen minutes and kept at -20 °C (29, 30).

***Metabolic factors analysis***

Biochemical analysis was conducted using a BT-3000 analyzer (Biotecnica Co., Italy) and standard kits for the measurement of serum creatinine (SCr), blood urea nitrogen (BUN), urine creatinine (UCr), and finally, triglyceride (TG), high-density lipoprotein cholesterol (HDL-c), and total cholesterol (TC). Low-density lipoprotein cholesterol (LDL-c) was calculated through the Fried Ewald equation (23). Additionally, the 24-hour urine creatinine (UCr) excretion was determined by collecting urine over 24 hr using metabolic cages. Urinary albumin levels were determined using an enzyme-linked immunosorbent immunoassay (ELISA) kit (Padtanelm, Tehran, Iran). An ELISA kit (Crystal Chem., Co., United States) and the sandwich enzyme immunoassay method were utilized to determine the serum insulin level.

***Glycation measurement: HbA***_1C_ ***and AGE***

HbA_1C_ levels (glycated hemoglobin) were determined using high-performance liquid chromatography (HPLC), which detects glucose-bound hemoglobin (31). The fluorimetry method was used to identify AGEs by diluting serum samples 1:50 with 0.1 sodium phosphate buffer and assessing their fluorescence intensity using a spectrofluorimeter (PerkinElmer Co., United States). The findings were expressed as the percentage of fluorescence emission (%F) (32). 

***Homogenization of kidney tissue***

To prepare the kidney tissue homogenate, a 100-milligram segment of kidney tissue was extracted from each rat and homogenized using liquid nitrogen, lysate buffer, and PMSF (phenylmethylsulfonyl fluoride) as an antiprotease agent.

***Measurement of oxidative stress and anti-oxidant markers Lipid peroxidation***

Lipid peroxidation in serum and kidney tissue homogenates was assessed by quantifying malondialdehyde (MDA) using the thiobarbituric acid method (33). Protein concentration was determined via the Bradford method, employing bovine serum albumin as the standard (34). Additionally, the level of protein carbonyl was analyzed following the spectrophotometric procedure detailed by Colombo *et al*. (35). This method involves the reaction of 2,4-Dinitroophenylhydrazine (DNPH) with carbonyl groups, resulting in the formation of a color adduct with absorption at a wavelength of 366 nm.

***Total anti-oxidant capacity (TAC)***

The assessment of TAC was conducted via the ferric-reducing anti-oxidant power (FRAP) method. In this method, anti-oxidants reduce Fe^3+-^tripyridyltriazine (TPTZ) to Fe^2+^ and form a blue-colored Fe II-TPTZ complex with absorption at a wavelength of 593 nm (36).

***Anti-oxidant enzyme activity and glutathione (GSH) level***

GSH levels and superoxide dismutase (SOD) activity were assessed following the guidelines provided by the kit manufacturer (Kalazist Co., Tehran, Iran). H_2_O_2_ was used to measure catalase enzyme activity in accordance with the methodology outlined in our previous investigation (37). The reaction between H_2_O_2_ and catalase was stopped by using ammonium molybdate, which resulted in a yellow-colored complex with absorption observed at a wavelength of 374 nm (38).

***NO level***

Nitrite and nitrate levels were measured as the index for nitric oxide (NO) formation based on the Griess method (39). 

***Gene expression***

To extract RNA, 100 mg of fresh tissue was utilized and homogenized by dissolving it in 1 ml of TRIzol on ice, followed by centrifugation for ten minutes at 12000 g and 4°C. Next, 200 ml of chloroform was introduced to the supernatant and centrifuged for fifteen minutes at 12000 g and 4 °C. Subsequently, 500 ml of isopropyl alcohol was added to the RNA-containing aqueous phase and centrifuged for five minutes at 7500 g and 4 °C. After washing the RNA pellet with 75% ethanol, it was centrifuged for five minutes at 7500 g and 4°C, then dissolved in 50 DEPC water. Quantitative RNA analysis was conducted using a NanoDrop (Thermo Scientific NanoDrop 2000c Spectrophotometer, USA), while qualitative analysis was carried out through electrophoresis on a 1% agarose gel. Following RNA extraction, complementary DNA (cDNA) was synthesized using a polymerase chain reaction (PCR) thermal cycler and a cDNA synthesis kit (Pars Tous Biotechnology, Iran). Details regarding primer sequencing and thermal cycling steps are available in our prior publication (32). 

***Kidney histological assessment***

The kidney tissue samples were rapidly fixed in 10% formaldehyde solution, followed by dehydration in alcohol, embedding in paraffin, slicing into 5-micron sections, and staining with hematoxylin and eosin for analysis. Subsequently, a histologist employed an Eclipse 80i optical microscope (magnification: 400x) to examine the slides for alterations in the mesangial matrix, focusing on proximal tubules, glomerular and tubular swelling, lymphocyte infiltration, and the presence of hyaline casts in urinary tubes. Based on changes in the renal cortex area, histopathological fibrosis and chronic nephropathy severity were scored on a 0–5 scale. The scale criteria were as follows: zero indicated normal tissue, one represented less than 1% involvement, two indicated involvement between 1% and 25%, three reflected involvement between 26% and 50%, four depicted involvement between 51% and 75%, and five indicated involvement between 76% and 100% of observed inflammatory and morphological alterations within the glomeruli and tubules, as outlined in reference (40).

***Data analysis***

Data analysis was conducted using SPSS software (version 27.0), employing one-way analysis of variance (ANOVA) and Tukey’s *post hoc* test, with a significance level set at *P*<0.05. Results are expressed as mean ± standard deviation.

## Results

***Metabolic factors***

At the conclusion of the eight-week WWI intervention, significant reductions were observed in FBS levels in the DW group compared to both their baseline readings and the DC group (*P*<0.05), as illustrated in Figure 1. Additionally, the prevalence of dyslipidemia among rats following the intervention, notably observed in the DC group, suggests alterations in lipid metabolism, as depicted in Figure 2. The concentrations of LDL-c, TC, and TG were notably higher in the DC group compared to the HC group, while HDL-c levels were significantly lower (*P*<0.05). Conversely, the DW group exhibited significantly lower LDL-c, TC, and TG levels, along with higher HDL-c levels, compared to the DC group (*P*<0.05).

At the conclusion of the WWI intervention, the DW group exhibited lower levels of SCr, BUN, and microalbuminuria, alongside significantly higher GFR levels compared to the DC group (*P*<0.05) (Figure 3). Additionally, the DC group displayed significantly higher levels of AGE compared to both the HC and DW groups (*P*<0.05). While insulin levels were higher in the DW group compared to the DC group, no significant difference was observed between the DW and DC groups regarding HbA1C levels (*P*>0.05). Furthermore, the DC group exhibited significantly lower levels of HSP70 compared to the HC, DW, and HW groups (*P*<0.05) (Figure 4).

***Oxidative stress biomarkers***

In assessing biochemical parameters, the intervention groups (DW, HW, and HC) consistently demonstrated favorable outcomes compared to the DC group (Figure 5). Marked reductions in oxidative stress were observed, as indicated by significantly lower concentrations of MDA and NO in both serum and kidney tissue (*P*<0.05). Additionally, anti-oxidant status was notably enhanced, with increased thiol group levels, specifically reduced glutathione (GSH), in serum and kidney tissue. The enzymatic anti-oxidant activities of SOD and catalase in serum and kidney tissue exhibited significant elevations in the intervention groups (*P*<0.05). Furthermore, the TAC levels in both serum and kidney tissue were markedly higher, indicating improved anti-oxidant defense mechanisms (*P*<0.05). Simultaneously, the intervention groups exhibited elevated levels of protein carbonylation (PC) in both serum and kidney tissue in comparison to the DC group (*P*<0.05) (Figure 5).

***Gene expression***

The DC group showed notably higher mRNA levels for the Transforming growth factor ß1 (TGFß1), Receptor for Advanced Glycation Endproducts (RAGE), and Vascular endothelial growth factor (VEGF) genes compared to the HC and DW groups (*P*<0.05). Additionally, real-time PCR analysis affirmed a significant increase in gene expression, particularly for TGFß1, RAGE, and VEGF, in the kidney tissue of the DC group compared to the HC and DW groups (*P*<0.05) (Figure 6).

***Histological changes of the kidney tissue***

Histological examination of kidney tissue revealed no morphological abnormalities in the HC and HW groups, with rats in these groups exhibiting regular glomerular morphology (Figure 6). Conversely, the DC group displayed significantly increased glomerular and tubular swelling, particularly in the proximal tubules, along with the presence of hyaline casts in urinary tubes and lymphocyte infiltration compared to the HC group. Additionally, while the mean score of tissue damage in the DW group was lower than that in the DC group (2.6±0.002 vs. 3.25±0.47), this difference did not reach statistical significance (*P*>0.05) (Figure 7, Table 1).

***Toxicity and mortality***

None of the rats in the HC and the HW groups experienced death, while 50% of rats in the DC and the DW groups died during the study. Moreover, none of the rats in the HW and the DW groups showed any symptoms of WWI toxicity or side effects such as alopecia, eczema, and behavioral changes.

## Discussion

This study explores the effects of WWI on inflammation, kidney function, and tissue damage in diabetic rats induced with STZ. The results highlight the connection between hyperglycemia and the development of DN, in line with previous research illustrating pathways where hyperglycemia contributes to complications such as the overproduction of advanced glycation end products (AGEs), free radical production, and induction of inflammation (41).

The Diabetic Warm Water Immersion (DW) group exhibited significant enhancements in kidney function parameters and oxidative stress biomarkers when compared to the Diabetic Control (DC) group (Figure 1-5). Prior studies supporting the positive influence of heat therapy on blood glucose, AGEs, and growth factors through increased Heat Shock Protein 70 (HSP70) levels align with these findings (25, 42). Heat therapy’s multifaceted impact involves reducing AGE levels, enhancing anti-oxidant capacity, inducing HSP70 production, and suppressing reactive oxygen species (ROS) production. The study underscores the nuanced response to heat therapy, revealing that while it increases chemical chaperones to prevent AGE aggregation and protein misfolding, it also induces free radical production, contributing to increased HSP70 levels (43). Continued treatment triggers protein kinase phosphorylation and activates protein kinase (AMPK), ultimately enhancing insulin signaling and sensitivity, and reducing inflammation in diabetic rats over time (44). Discrepancies with a study demonstrating a reduction in HSP70 levels after heat adaptation are attributed to variations in WWI parameters (42).

In the DW group, reduced tissue and serum levels of oxidative stress markers such as MDA and NO, and elevated levels of anti-oxidant markers like TAC, GSH, SOD, and catalase, indicate the positive effects of WWI on oxidative stress and anti-oxidant markers (Figure 5). Oxidative stress is a critical mechanism in DN induction, potentially leading to apoptosis, reduced kidney mass, and DN. The study emphasizes the balance between oxidative stress and anti-oxidant defense mechanisms, showing that SOD and catalase contribute to increase TAC, thereby reducing oxidative stress factors and potentially limiting kidney tissue damage in diabetic rats (45, 46).

Genetic analyses unveiled significant up-regulation of TGFß1 and RAGE genes in the DC group, suggesting a direct correlation between diabetes and increased expression of these genes. This aligns with existing research highlighting elevated TGFß1 gene expression in nephropathy and the impact of AGEs binding to their receptors in up-regulating TGFß1 and RAGE gene expressions. Activation of TGFß1 results in collagen synthesis and abnormal extracellular matrix increase, associated with clinical manifestations of DN (47-51). In contrast, the DW group exhibited significantly lower expression of growth factor genes, indicating potential protective effects of WWI against glycation-related alterations. Some studies have shown that interventions like WWI help deter glycation and cross-linking processes, leading to a decrease in the expression of RAGE and TGFß1 genes in kidney tissues (52, 53). The process of kidney damage was found to be slowed down in DN-afflicted rats by using RAGE antibody and gene deletion in further studies (54, 55).

In the DC group, the expression of the VEGF gene in kidney tissue was notably higher compared to both the Healthy Control (HC) and DW groups (Figure 6). This suggests that diabetes amplifies VEGF gene expression, whereas WWI diminishes it. VEGF is crucial in orchestrating vascular system responses and advancing endothelial cell dysfunction in complications related to DM. The study suggests that WWI may mitigate these effects by reducing blood glucose, AGEs, and signaling pathways triggered by the AGE/RAGE reaction, ultimately decreasing protein kinase C enzyme activity and VEGF expression.

The histological evaluation in this study revealed an increase in mesangial matrix, as well as glomerular and tubular swelling, particularly evident in the proximal tubules, alongside the presence of hyaline casts and lymphocyte infiltration in kidney tissue in the DC group (refer to Figure 7). While these tissue changes were less pronounced in the DW group compared to the DC group, the difference was not statistically significant. Research indicates that advanced glycation end products (AGEs) can result in thickening of the glomerular basement membrane, augmented extracellular matrix, diminished elasticity of glomerular capillaries, heightened glomerular filtration and permeability, increased proteinuria, and damage to glomerular capillaries, ultimately leading to diabetic nephropathy (56, 57).

 It seems that by reducing blood glucose and AGEs, WWI reduces the expression of the TGFß1 and the VEGF genes and prevents kidney tissue damage. While providing valuable insights, the study acknowledges limitations, including the absence of measurements for gene expression of NF-κB and Heat Shock Proteins (HSPs), as well as interleukins as inflammatory factors. Future studies are recommended to address these factors, enhancing our understanding of WWI effects in diabetic rats.

## Conclusion

WWI is an inexpensive and effective intervention to alleviate the symptoms of DM and DN. Of course, further studies are still needed to produce more conclusive evidence in this area.

## Data Availability

The authors confirm that the data supporting the findings of this study are available within the article.

## References

[1] Gohda T, Mima A, Moon JY, Kanasaki K (2014). Combat diabetic nephropathy: From pathogenesis to treatment. J Diabetes Res.

[2] Ahmed N (2005). Advanced glycation endproducts-role in pathology of diabetic complications. Diabetes Res Clin Pract.

[3] Zou J, Yu X, Qu S, Li X, Jin Y, Sui D (2014). Protective effect of total flavonoids extracted from the leaves of Murraya paniculata (L. ) Jack on diabetic nephropathy in rats. Food Chem Toxicol.

[4] Al-Kafaji G, Golbahar J (2013). High glucose-induced oxidative stress increases the copy number of mitochondrial DNA in human mesangial cells. Biomed Res Int.

[5] Papadopoulou-Marketou N, Chrousos GP, Kanaka-Gantenbein C (2017). Diabetic nephropathy in type 1 diabetes: A review of early natural history, pathogenesis, and diagnosis. Diabetes Metab Res Rev.

[6] Rahbar S, Figarola JL (2003). Novel inhibitors of advanced glycation endproducts. Arch Biochem Biophys.

[7] Daroux M, Prévost G, Maillard-Lefebvre H, Gaxatte C, D’Agati VD, Schmidt AM (2010). Advanced glycation end-products: implications for diabetic and non-diabetic nephropathies. Diabetes Metab.

[8] Mason RM, Wahab NA (2003). Extracellular matrix metabolism in diabetic nephropathy. J Am Soc Nephrol.

[9] Oldfield MD, Bach LA, Forbes JM, Nikolic-Paterson D, McRobert A, Thallas V (2001). Advanced glycation end products cause epithelial-myofibroblast transdifferentiation via the receptor for advanced glycation end products (RAGE). J Clin Invest.

[10] Saraheimo M, Teppo AM, Forsblom C, Fagerudd J, Groop PH (2003). Diabetic nephropathy is associated with low-grade inflammation in Type 1 diabetic patients. Diabetologia.

[11] Shah SV, Baliga R, Rajapurkar M, Fonseca VA (2007). Oxidants in chronic kidney disease. J Am Soc Nephrol.

[12] Wu XQ, Zhang DD, Wang YN, Tan YQ, Yu XY, Zhao YY (2021). AGE/RAGE in diabetic kidney disease and ageing kidney. Free Radic Biol Med.

[13] Yokoyama H, Araki S, Kawai K, Hirao K, Oishi M, Sugimoto K (2015). Pioglitazone treatment and cardiovascular event and death in subjects with type 2 diabetes without established cardiovascular disease (JDDM 36). Diabetes Res Clin Pract.

[14] Zhang S, Xu H, Yu X, Wu Y, Sui D (2017). Metformin ameliorates diabetic nephropathy in a rat model of low-dose streptozotocin-induced diabetes. Exp Ther Med.

[15] Hoogeveen EK (2022). The Epidemiology of Diabetic Kidney Disease. Kidney and Dialysis.

[16] Li S, Xie H, Shi Y, Liu H (2022). Prevalence of diabetic nephropathy in the diabetes mellitus population: A protocol for systematic review and meta-analysis. Medicine (Baltimore).

[17] Diamant S, Eliahu N, Rosenthal D, Goloubinoff P (2001). Chemical chaperones regulate molecular chaperones in vitro and in cells under combined salt and heat stresses. J Biol Chem.

[18] Fleshner M, Johnson JD (2005). Endogenous extra-cellular heat shock protein 72: Releasing signal(s) and function. Int J Hyperthermia.

[19] Laplante AF, Moulin V, Auger FA, Landry J, Li H, Morrow G (1998). Expression of heat shock proteins in mouse skin during wound healing. J Histochem Cytochem.

[20] Welch WJ, Brown CR (1996). Influence of molecular and chemical chaperones on protein folding. Cell Stress Chaperones.

[21] Guzhova I, Margulis B (2006). Hsp70 chaperone as a survival factor in cell pathology. Int Rev Cytol.

[22] Tan AL, Forbes JM, Cooper ME (2007). AGE, RAGE, and ROS in diabetic nephropathy. Semin Nephrol.

[23] Kurucz I, Morva A, Vaag A, Eriksson KF, Huang X, Groop L (2002). Decreased expression of heat shock protein 72 in skeletal muscle of patients with type 2 diabetes correlates with insulin resistance. Diabetes.

[24] Yamagishi N, Nakayama K, Wakatsuki T, Hatayama T (2001). Characteristic changes of stress protein expression in streptozotocin-induced diabetic rats. Life Sci.

[25] Bathaie SZ, Jafarnejad A, Hosseinkhani S, Nakhjavani M (2010). The effect of hot-tub therapy on serum Hsp70 level and its benefit on diabetic rats: A preliminary report. Int J Hyperthermia.

[26] Rakonczay Z Jr, Takács T, Mándi Y, Iványi B, Varga S, Pápai G (2001). Water immersion pretreatment decreases pro-inflammatory cytokine production in cholecystokinin-octapeptide-induced acute pancreatitis in rats: possible role of HSP72. Int J Hyperthermia.

[27] Yang FL, Lee CC, Subeq YM, Lee CJ, Ke CY, Lee RP (2017). Heat adaptation from regular hot water immersion decreases proinflammatory responses, HSP70 expression, and physical heat stress. J Therm Biol.

[28] Ghasemi A, Jeddi S (2023). Streptozotocin as a tool for induction of rat models of diabetes: A practical guide. Excli J.

[29] Skovsø S (2014). Modeling type 2 diabetes in rats using high fat diet and streptozotocin. J Diabetes Investig.

[30] Tesch GH, Allen TJ (2007). Rodent models of streptozotocin-induced diabetic nephropathy. Nephrology (Carlton).

[31] Marshall SM, Barth JH (2000). Standardization of HbA1c measurements: a consensus statement. Ann Clin Biochem.

[32] Jozi F, Kheiripour N, Taheri MA, Ardjmand A, Ghavipanjeh G, Nasehi Z (2022). L-lysine ameliorates diabetic nephropathy in rats with streptozotocin-induced diabetes mellitus. Biomed Res Int.

[33] Janero DR (1990). Malondialdehyde and thiobarbituric acid-reactivity as diagnostic indices of lipid peroxidation and peroxidative tissue injury. Free Radic Biol Med.

[34] Bonjoch NP, Tamayo PR, Reigosa Roger MJ (2001). Protein Content Quantification by Bradford Method. Handbook of Plant Ecophysiology Techniques.

[35] Colombo G, Clerici M, Garavaglia ME, Giustarini D, Rossi R, Milzani A (2016). A step-by-step protocol for assaying protein carbonylation in biological samples. J Chromatogr B Analyt Technol Biomed Life Sci.

[36] Benzie IF, Strain JJ (1996). The ferric reducing ability of plasma (FRAP) as a measure of “anti-oxidant power”: The FRAP assay. Anal Biochem.

[37] Kabiri-Arani S, Motallebi M, Taheri MA, Kheiripour N, Ardjmand A, Aghadavod E (2024). The effect of heat-killed lactobacillus plantarum on oxidative stress and liver damage in rats with bile duct ligation-induced hepatic fibrosis. Probiotics Antimicrob Proteins.

[38] Hajian H, Motallebi M, Akhavan Taheri M, Kheiripour N, Aghadavod E, Shahaboddin ME (2024). The preventive effect of heat-killed Lactobacillus plantarum on male reproductive toxicity induced by cholestasis in rats. Food Chem Toxicol.

[39] Shangari N, O’Brien PJ (2006). Catalase activity assays. Curr Protoc Toxicol.

[40] Nasehi Z, Kheiripour N, Taheri MA, Ardjmand A, Jozi F, Shahaboddin ME (2023). Efficiency of hesperidin against liver fibrosis induced by bile duct ligation in rats. Biomed Res Int.

[41] Zhang Q, Davis KJ, Hoffmann D, Vaidya VS, Brown RP, Goering PL (2014). Urinary biomarkers track the progression of nephropathy in hypertensive and obese rats. Biomark Med.

[42] Remuzzi G, Schieppati A, Ruggenenti P (2002). Clinical practice Nephropathy in patients with type 2 diabetes. N Engl J Med.

[43] Zhang H, Gong W, Wu S, Perrett S (2022). Hsp70 in Redox Homeostasis. Cells.

[44] Jafarnejad A, Bathaie SZ, Nakhjavani M, Hassan MZ (2008). Effect of spermine on lipid profile and HDL functionality in the streptozotocin-induced diabetic rat model. Life Sci.

[45] Forghani N, Karimi Z, Mokhtari M, Shariati M, Masjedi F (2023). Association of oxidative stress with kidney injury in a hyperandrogenemic female rat model. Iran J Med Sci.

[46] Vona R, Pallotta L, Cappelletti M, Severi C, Matarrese P (2021). The impact of oxidative stress in human pathology: Focus on gastrointestinal disorders. Antioxidants (Basel).

[47] Kanwar YS, Wada J, Sun L, Xie P, Wallner EI, Chen S (2008). Diabetic nephropathy: mechanisms of renal disease progression. Exp Biol Med (Maywood).

[48] Khalid M, Petroianu G, Adem A (2022). Advanced glycation end products and diabetes mellitus: mechanisms and perspectives. Biomolecules.

[49] Pourghasem M, Shafi H, Babazadeh Z (2015). Histological changes of kidney in diabetic nephropathy. Caspian J Intern Med.

[50] Ward DT, Yau SK, Mee AP, Mawer EB, Miller CA, Garland HO (2001). Functional, molecular, and biochemical characterization of streptozotocin-induced diabetes. J Am Soc Nephrol.

[51] Ziyadeh FN (2004). Mediators of diabetic renal disease: the case for tgf-Beta as the major mediator. J Am Soc Nephrol.

[52] Lu L, Peng WH, Wang W, Wang LJ, Chen QJ, Shen WF (2011). Effects of atorvastatin on progression of diabetic nephropathy and local RAGE and soluble RAGE expressions in rats. J Zhejiang Univ Sci B.

[53] Nakamura S, Li H, Adijiang A, Pischetsrieder M, Niwa T (2007). Pyridoxal phosphate prevents progression of diabetic nephropathy. Nephrol Dial Transplant.

[54] Flyvbjerg A, Denner L, Schrijvers BF, Tilton RG, Mogensen TH, Paludan SR (2004). Long-term renal effects of a neutralizing RAGE antibody in obese type 2 diabetic mice. Diabetes.

[55] Myint KM, Yamamoto Y, Doi T, Kato I, Harashima A, Yonekura H (2006). RAGE control of diabetic nephropathy in a mouse model: Effects of RAGE gene disruption and administration of low-molecular weight heparin. Diabetes.

[56] Forbes JM, Cooper ME, Oldfield MD, Thomas MC (2003). Role of advanced glycation end products in diabetic nephropathy. J Am Soc Nephrol.

[57] Tanji N, Markowitz GS, Fu C, Kislinger T, Taguchi A, Pischetsrieder M (2000). Expression of advanced glycation end products and their cellular receptor RAGE in diabetic nephropathy and nondiabetic renal disease. J Am Soc Nephrol.

